# Women’s empowerment and infant mortality in Latin America: evidence from 286 cities

**DOI:** 10.1080/23748834.2021.1908794

**Published:** 2021-04-22

**Authors:** Ana Ortigoza, Ariela Braverman, Philipp Hessel, Vanessa Di Cecco, Amélia Augusta Friche, Waleska Teixeira Caiaffa, Ana V. Diez Roux

**Affiliations:** a Urban Health Collaborative, Drexel University, Philadelphia, PA, USA; b Escuela de Gobierno Alberto Lleras Camargo, Universidad de los Andes, Bogotá, Colombia; c Instituto Salud Colectiva, Universidad Nacional de Lanus, Buenos Aires, Argentina; d Observatório de Saúde Urbana em Belo Horizonte, Universidade Federal de Minas Gerais, Belo Horizonte, Brazil

**Keywords:** Women’s empowerment, infant mortality, Latin America

## Abstract

Levels of women’s empowerment (WE) can contribute to differences in infant mortality rates (IMRs) across cities. We used a cross-sectional multilevel study to examine associations of WE with IMRs across 286 cities in seven Latin American countries. We estimated IMRs for 2014–2016 period and combined city socioeconomic indicators into factors reflecting living conditions and service provision. WE was operationalized: (1) in cities, by using scores for women’s labor force participation (WLFP) and educational attainment among women derived from education and employment indicators disaggregated by sex; (2) in countries, by including a scale of enforcements of laws related to women’s rights. We estimated adjusted percent differences in IMRs associated with higher WE scores across all cities and stratified by country GDP. We found substantial heterogeneity in IMRs and WE across cities. Higher WLFP was associated with lower IMRs. Higher women’s educational attainment was associated with lower IMRs only in cities from countries with lower GDP. Poorer national enforcement of laws protecting women’s rights was associated with higher IMRs in all countries. Women’s empowerment could have positive implications for population health. Fostering women’s socioeconomic development and girls’ education should be part of strategies to reduce IMRs in cities of Global South.

## Background

As urban populations worldwide continue to grow, it has become increasingly important to identify how urban policies and programs can be leveraged to improve health in cities (Ezzati *et al*. 2018). Infant mortality is a key indicator of population health. Despite past evidence that infant mortality rates (IMRs) are lower in urban than in rural areas (de Carvalho and Wood 1978) important differences in IMRs exist both between and within cities (Kimani-Murage *et al*. 2014). Few studies have directly examined variations on IMRs across cities and its predictors.

A critical factor in promoting infant health is the status of women (Varkey and Lesnick 2010). Higher educational attainment in women of reproductive age has been linked to a reduction in IMRs at the country level, through reductions in adolescent birth rates and premature births (Gakidou *et al*. 2010), and through improvement in women’s skills as caregivers (Heaton 2015). Women with more financial autonomy appear to allocate more resources to their children’s health (Pratley 2016). In addition to effects on childbearing and childrearing, women’s social status is linked to women’s participation in political decision-making which has positive impacts on population levels of education (Clots-Figueras 2012) child welfare (Quamruzzaman and Lange 2016, Hessel *et al*. 2020), and the formulation of social policies that benefit living conditions in children (UN 2005).

Actions supporting women’s development have not taken place in the same way across countries and cities. Although women in cities are more likely to perceive some advantages over their rural counterparts in terms of employment and education (ECLAC 2016), women from lower socio- economic positions, in particular, remain more economically vulnerable and are more susceptible to ambient risks present in marginalized areas in urban settings, as they are more likely to remain at home or work near the place where they live (Chant 2013).

To date, evidence on effects of women’s empowerment on child’s health have been described mostly at national levels and involving cross-countries comparison (Pratley 2016, Macmillan *et al*. 2018). These approaches, however, are limited in accounting for the heterogeneity in women’s empowerment across what are often very diverse urban areas.

Latin America with its very high levels of urbanization and its many diverse cities provides a unique opportunity to understand the contributions of women’s empowerment to differences in IMRs across urban areas. The region has recently experienced significant emergence in feminist grass-roots movements in response to the unequal socioeconomic conditions and access to resources that women experience, particularly in large urban areas (Gasparini and Marchionni 2015). In this study we examine whether differences in measures of women’s empowerment are related to variability in IMRs across cities in Latin America and whether these relationships persist after accounting for other predictors of infant mortality in cities.

## Methods

### Sample

Data were drawn from the SALURBAL project, which included a total of 371 cities of 100,000 or more inhabitants in 2010 in 11 countries. Cities were defined as a single administrative unit or combination of adjacent administrative units (i.e. municipios, comunas, partidos, delegaciones, cantones, or corregi-mientos) that were part of the urban extent as determined from satellite imagery (Quistberg *et al*. 2019). For this study, we included cities for which vital statistics registries were available for 2014 to 2016 and had good quality death registrations (mortality coverage >90%) based on a separate analysis of adult mortality (Bilal *et al*. 2019). Of the 371 cities, 11 cities (5 cities in Nicaragua, and 3 cities in El Salvador and Guatemala, respectively) were excluded due to lack of vital statistics registries for the years of study; and 74 cities (9 cities in Brazil, 19 in Colombia, 31 in Mexico and 15 in Peru) were excluded because of mortality coverage <90%, leaving for analysis 286 cities located in Argentina, Brazil, Chile, Colombia, Costa Rica, Mexico, Peru, and Panama. Cities with inadequate mortality coverage had poorer living conditions and lower provision of water connected to public network compared to cities included in this study (see Supplementary material).

### Outcome

*Infant mortality rates (IMRs)*. We calculated IMRs (deaths less than 1 year of age per 1000 live births) for the period 2014–2016. Deaths and live births were retrieved based on deceased’s and maternal place of residence, respectively. Three years were pooled to increase stability of the estimates.

### Exposures

#### Women’s empowerment (WE)

WE in cities was operationalized by retrieving indicators of education and employment disaggregated by sex from national censuses. We retrieved a total of 8 socioeconomic indicators and conducted principal component analyses (PCA) in order to identify distinct sets that could be combined into scores. The PCA identified two different domains that incorporated 6 of the 8 indicators. Variables related to school attendance were dropped as they were not related to the rest of the variables. Each indicator was standardized to a mean of zero and standard deviation (SD) of one and values for different indicators were added together to create two women’s empowerment scores:

- Women’s labor force participation (WLFP), which describes aspects of women’s socioeconomic position and also accounts for gender inequalities in education and employment (Howe *et al*. 2012, Chant 2013, ECLAC 2016). It includes the female-to-male ratio of the proportion of population aged 25 or older who completed secondary education or above, and the female-tomale ratio of the proportion of the population who completed university degree or above; labor force participation among females 15 years or older; and the ratio of the labor force participation rate among females to the labor force participation rate among males. Higher score values signify greater participation of women in labor force and greater education of women relative to men.
- Educational attainment among women (EAW) which includes the proportion of the female population aged 25 or older with complete high school or above and the proportion of the female population aged 25 year or older with complete university degree or above. Higher score values signify greater education achievement among women.

We also included a scale developed by the Women’s Stats Project measuring the degree to which countries have and enforce laws supporting women’s rights (country law enforcement scale or CLE scale), including education, family, and physical security (Women Stats Projects-Codebook 2019). We included the scale for year 2015. Values range from 0 to 4, with lower values signifying higher level of country enforcement.

### Other covariates

#### Urban social environment

We included two urban social environment scores also based on PCA on a total of eight variables that were identified to be related with infant mortality in previous work (Ortigoza *et al*. 2020).

- Score of living conditions which includes (1) percentage of household with piped water inside the dwelling, as a marker of house conditions; (2) percentage of households with overcrowding conditions (more than 3 people per room, excluding kitchen and bathroom); and (3) percentage of population aged 15–17 attending school, as a marker of social marginalization, since low school attendance among adolescents has been linked to poverty and early participation in the labor market, as well as exclusion from productive systems (Cardenas *et al*. 2015, Bodenhorn 2006), We reverse coded the overcrowding indicator so higher score values signify better living conditions and lower poverty levels.
- Score of service provisions, which include public services that cities provide to dwellings, include (1) percent of households with access to water source from a municipal public or private water network and (2) percentage of households with sewage system connected to a municipal public or private sewage network. Higher score values signify better service provision in cities.

#### Vaccine coverage in infant population

City coverage of first dose of triple viral vaccine (MMR1, measles-mumps-rubella vaccine) among the population of children 1 year of age was used as a proxy of health care access among infants. MMR1 presents a schedule that is similar across countries and made it suitable for harmonization (PAHO 2019). Data for year 2016 was provided by World Health Organization (WHO 2019).

#### National gross domestic product per capita (GDP per capita)

We retrieved the national Real GDP (output-based) per population for 2015 for each country from Penn World Tables (Feenstra *et al*. 2019) and use the median value of the sample (15,530.7 US$) to categorize countries in two groups: above and below the median GDP per capita.

### Statistical analysis

We first described variations in IMRs, and WE measures across cities within countries as well as the distribution of city level covariates across categories of city-level measures of WE. We then estimated the association of women’s empowerment predictors with IMRs using Poisson multilevel models (cities nested within countries). Each WE predictor was first explored separately and then included in a multivariable model with all predictors together. In order to determine associations of women’s empowerment measures with IMRs independently from other city-characteristics, we adjusted the final model for SE scores and MMR1 coverage. We explored effect modification by country GDP per capita, by repeating the same modelling sequence in countries with GDP per capita above and below the median value of the sample, and then by testing interactions between GDP per capita and city-level predictors.

## Results

**Figure 1:**
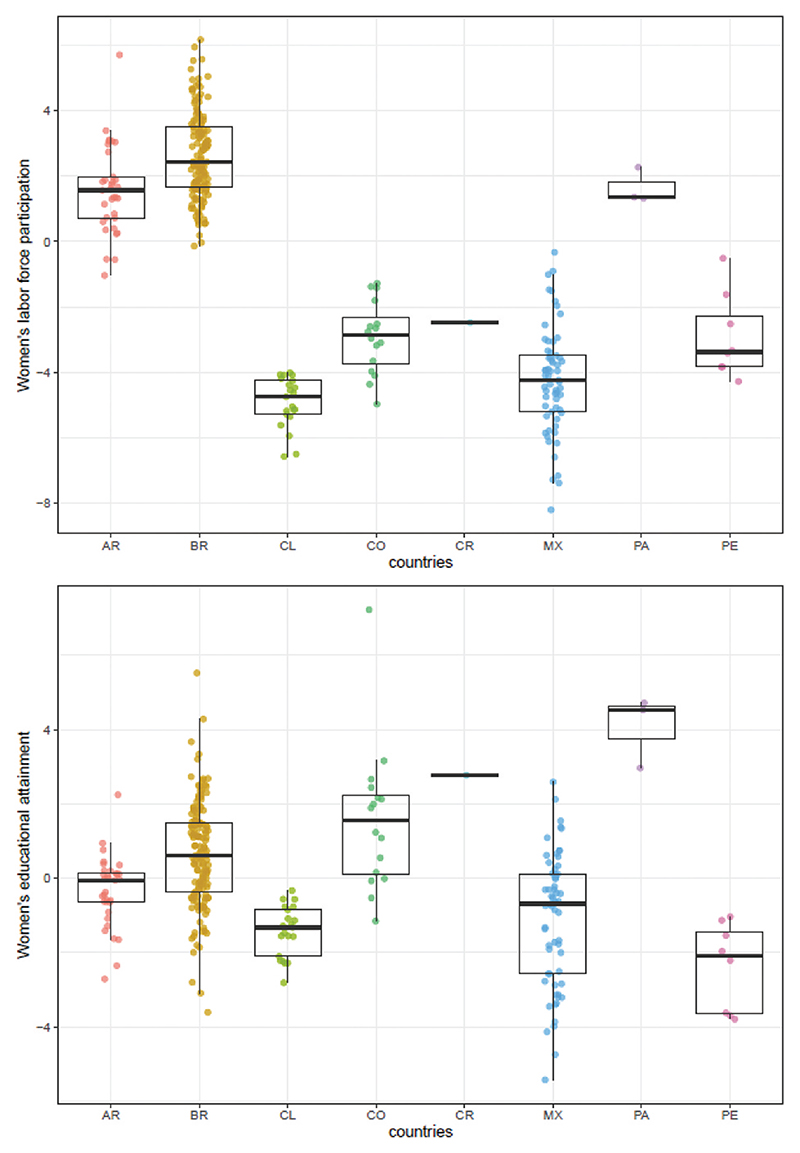
Distribution of women’s empowerment in cities by country (n = 286 cities). Each dot represents a city-estimate of women’s labor force participation score (above) and women’s educational attainment score (below), and boxplots show its country median and range distribution. Scores were created based on z-score indicators, so that zero value represents the mean value of each measure. Ref: AR = Argentina; BR = Brazil, CL = Chile; CO = Colombia; CR = Costa Rica; MX = Mexico; PA = Panama; PE = Peru.

Figure 1 shows the distribution of women’s empowerment measures across cities. Women’s empowerment scores varied across countries but there was also substantial variation across cities within each country. For IMRs, the greatest heterogeneity is observed across cities within countries. The intra class correlation (ICC) was 0.43 implying that 57% of the total variance in IMRs was across cities within countries. For women’s empowerment measures, differences across countries were greater for women’s labor force participation score (ICC = 0.83) than for women’s educational attainment score (ICC = 0.63).

Selected characteristics of cities included in analyses by quartiles of women’s labor force participation score are shown in Table 1. All cities in Chile had levels of women’s labor force participation in the lowest quartile and all cities in Costa Rica and Panama were in middle quartiles. Cities in Colombia, Mexico and Peru were in the middle- to lower quartiles of women’s labor force participation while cities in Argentina and Brazil were in the mid -higher quartiles. Cities with low levels of women’s labor force participation had significantly lower proportion of population with adequate living conditions compared to the rest of the cities (Table 1). Cities in the lowest quartile of women’s labor force participation also showed significantly lower mean levels of women’s education than other cities: 32.8% and 9.4% for complete high school or more and complete university or more, respectively, compared to around 40% and 13% in the rest of the cities. Mean level of MMR1 coverage in the sample was 91.3% and did not differ by quartiles of women’s labor force participation. Mean IMR was 11.2 deaths per 1000 live births and IMRs were lowest in the lowest quartile of women’s labor force participation (Table 1).

Table 2 shows percent differences in IMRs associated with a one SD higher WE and SE scores, one-unit higher CLE scale, and 1% higher MMR1 vaccine coverage. In the unadjusted analyses, only the score of educational attainment among women and CLE scale showed significant associations with IMRs (models 2 and 3). In the final model (model 5), a higher score of women’s labor force participation and a higher value on the CLE scale were both significantly associated with IMRs: a one SD higher score of women’s participation in labor force was associated with 6.1% (95% CI −11.1 –0.8%) lower IMRs, and a one unit higher in CLE scale (less enforcement) was associated with a 16.6% (95% CI 3.6 31.4) higher IMR, after accounting for SE scores and MMR1 coverage (model 5). Better living conditions and better service provision were also associated with lower IMRs in the full model. The country random intercept remained statistically significant in all models suggesting the persistence of unexplained variation across countries even after city level factors were accounted for (Table 2). City-level factors explained part of the variability in IMRs across countries, as evidenced by a decrease in the country variance from 0.04 in the null model to 0.02 in the final model (model 5).

When countries were grouped by the value of GDP per capita (Table 3), higher scores of women’s labor force participation were associated with lower IMRs in countries below and above the GDP per capita median. The association was stronger in countries above the median GDP per capita, but the interaction was not statistically significant. Higher scores for living conditions were significantly and similarly associated with lower IMRs in countries above and below the median GDP per capita. In contrast, women’s educational attainment and service provision were only associated with lower IMRs in countries with GDP per capita below the median: the percent difference in IMRs per one SD higher score was -3.6% (95% CI -6.3 -0.9) for women’s educational attainment and -12.6% (95% CI -13.5 -11.7) for service provision (Table 3).

## Discussion

In this study of 286 Latin American cities, we found significant heterogeneity across cities in both IMRs and women’s empowerment. Higher women’s participation in the work force was linked to lower IMRs, independently of the level of health care coverage and socioeconomic conditions. Lower enforcement of laws related to women’s rights was related to higher IMRs. These associations were observed in countries below and above the median GDP per capita. However, GDP per capita modified the associations between IMRs and women’s educational attainment, such that a significant and negative association with IMRs was observed only in countries with GDP per capita below the median.

We found that heterogeneity in women’s empowerment measures across cities within countries was greater for women’s educational attainment than for women’s labor force participation. Women’s participation in labor force could be influenced by cultural and gender – role patterns that may differ more across countries than across cities within countries. The great heterogeneity observed in women’s educational attainment and in IMRs across cities within countries highlights the importance of examining features of cities as drivers of both access to women’s education (Beall 1996) and IMRs (Ortigoza *et al*. 2020).

In our study a one SD higher score of women’s labor force participation was associated with 6% lower IMRs. Similar associations have been reported in the United States: state measures of women’s employment and earnings were associated with lower infant mortality and teen birth rates after accounting for income inequality and state racial composition (Koenen *et al*. 2006). To our knowledge, ours is the first study to investigate these associations at the city level and across a large sample of cities in lower and middle-income countries.

A higher participation of women in the labor market has been described as an important contributor to the reduction of poverty and inequality in Latin America, particularly in urban areas where there is an increasing proportion of women-headed households (Gasparini and Marchionni 2015, ECLAC 2016). It has been posited that women with access to paid work and corresponding income make decisions regarding household expenditures and savings that are more oriented to children, which eventually contribute to better nutrition, care, and longterm human capital accumulation (Smith *et al*. 2004, Gasparini and Marchionni 2015). The increasing participation of women in the labor market has also been linked to decreasing natality and fertility which are also associated with lower infant mortality (Hojman 1992, Gakidou *et al*. 2010). This decline in natality and fertility is also a central feature of the progressive emancipation of women from a narrowly defined role in domestic and reproductive life (Chant 2013, Soto-Villagrán 2014).

In the full sample, educational attainment among women was associated with IMRs in the expected direction but the association disappeared when SE scores linked to living conditions and service provision were added to the model. However, analyses stratified by country GDP per capita showed that higher educational attainment among women was associated with lower IMRs even after adjustment for SE indicators in countries with GDP per capita below the sample median. It is possible that in countries with lower GDP per capita, women’s access to education remains an important barrier, possibly limiting labor force participation and its favorable effects (Howe *et al*. 2012). It is also possible that in countries with higher GDP per capita the measure of women’s education that we used may not capture differences in women’s circumstances relevant to IMRs after labor force participation is accounted for.

We showed that poor enforcement of laws concerning women’s right at a national level was associated with higher infant mortality: one-point higher CLE scale was associated with almost a 17% higher IMR. This adds to the evidence that fostering women’s development and improving women’s status may bring benefits beyond women’s own interests (Varkey and Lesnick 2010, Clots-Figueras 2012). These findings support the importance of actively engaging women in urban politics and governance to assure sustainability of this progress (ECLAC 2016, Hessel *et al*. 2020).

Some strengths of the study include the extensive compilation and harmonization of data on women’s empowerment and IMRs across a wide range of cities in Latin America and the incorporation of country as well as city level effects via multilevel modelling. Findings from this work support the importance of considering indicators of women and child’s health as part of the measures used for monitoring the achievements derived from women’s economic empowerment (Buvinic *et al*. 2020).

Although we included two important measures of women’s empowerment, we were not able to characterize other aspects of WE such as political participation of women in local and national governments which have been shown to be related to infant mortality in the region (Hessel *et al*. 2020). Due to data limitation we were not able to characterize the proportion of women living in poverty or the level of women’s income in cities. However, we showed that cities with lower levels of women’s labor force participation had also significantly lower proportion of population with adequate living conditions, suggesting the connection between urban poverty and low access to work and economic opportunities for women. Our measure of labor force participation does not capture the types of jobs that women are employed in or the benefits that their jobs may confer. Future work needs to further explore the intersection of women’s employment and income, education, and political participation as predictors of IMRs since opportunities for progress related to labor force participation (as well as the health consequences of employment itself) may be different for poor women or women with low education compared to wealthier women and women with higher education (UN 2017). Women with less education living in poor urban areas are more likely to have informal employments and hence they are more sensitive to economic downturns (UN 2017). Low wage jobs even in the formal economy can have adverse health and social impacts. Limited mobility and safety conditions in marginalized areas may constrain opportunities for better jobs or for job advancement (UN 2017). Indicators of women’s empowerment included in this study relied on census data and hence focused on access to resources for women’s empowerment such as eductation or employment. We were not able to include indicators related to other important domains of women’s empowerment such decision-making power (i.e. control over household expenditure), gender-based violence or measures of the empowering process itself (Buvinic *et al*. 2020) (i.e. self and collective-esteem), considered by many feminist scholars to be key aspects of women’s empowerment (Cornwall and Anyidoho 2010, Cornwall 2014). As future research continue to explore how women’s development in cities contributes to population’s health, more data need to be available at sub-national levels in order to better characterize the progress of women in urban areas.

Other limitations include the cross-sectional and ecological design and the use of a measure of vaccine coverage as a proxy of health-care access. Previous studies have shown that one-time and low complexity interventions, like measles vaccine coverage campaigns, tend to be more equally distributed in a population than specific high-skilled interventions (Barros and Victora 2013). A more accurate depiction of health-care coverage in the infant population may require the combination of several indicators related to family planning, antenatal care, multiple vaccine coverages and access to treatment during episodes of disease like diarrhea or pneumonia (Barros *et al*. 2012).

Under-registration of deaths is always a concern in analyses of infant mortality (Ribotta 2016). We addressed this issue in part by restricting the study to cities with good quality of data based on adult mortality estimations. However, it is possible that registration of infant deaths and live births is influenced by factors different from those that affect under-registration of adult mortality. If more underegistration of infant deaths is associated with lower women’s empowerment, the associations we report may be underestimates of true associations.

## Conclusion

As urbanization continues to increase all over the world, it is critical to identify urban policies that can be most impactful in reducing infant deaths, which remain high even in many urban areas. Our results illustrate the potential benefits that fostering women’s development, may bring to child wellness in cities. Strategies involving the education of girls and increasing employment opportunities for women need to be considered and coordinated with other cost-effective interventions already implemented in the region (such as perinatal care & skilled birth, vaccine delivery, nutritional supplements, oral rehydration and antibiotics therapy) as part of programs that promote child health. In addition, women and children need to count as priority beneficiaries of urban planning interventions, as they represent a large proportion of the urban poor in many cities from low and middle-income countries. More systematic data on women’s socioeconomic achievement as well as on indicators measuring the process of women’s empowerment will be needed at the city-level in order to assess and monitor the implementation of these strategies and their impacts.

## Supplementary Material

Table S1

## Figures and Tables

**Table 1:** City demographic and socio-economic characteristics by quartiles of women’s labor force participation score (n = 286 cities).

	Total	Women’s Labor Force Participation Score			p-value
	n (% of sample) or mean (SD)	<P25	P25-P75	>P75
Overall number of cities n (%)	286	71 (24.8%)	144 (50.3%)	71 (24.8%)	
Number of cities by country n (col%)					
Argentina	33 (11.5%)	–	26 (18.1%)	7 (9.9%)	
Brazil	143 (50.4%)	–	79 (54.9%)	64 (90.1%)	
Chile	21 (7.3%)	21 (29.6%)	–	–	
Colombia	16 (5.6%)	5 (7.0%)	11 (7.6%)	–	
Costa Rica	1 (0.3%)	–	1 (0.7%)	–	
México	61 (21.3%)	42 (59.2%)	19 (13.2%)	–	
Panama	3 (1.0%)	–	3 (2.1%)	–	
Peru	8 (2.8%)	3 (4.2%)	5 (3.5%)	–	
**Socio-economic predictors by domain, mean**					
**(SD)**					
Living conditions					
% of households with piped water in the	89.8 (11.6)	81.5 (15.6)	92.1 (8.6)	93..3 (7.3)	<0.0001
dwelling					
% of households with overcrowding in the house ^a^	4.8 (3.9)	8.0 (4.4)	4.2 (3.3)	2.9 (2.2)	<0.0001
% of population 15-17 attending school	80.3 (7.5)	73.9 (8.9)	81.8 (6.4)	83.6 (3.4)	<0.0001
Score	−0.04 (0.7)	−0.61 (0.7)	0.09 (0.6)	0.26 (0.3)	<0.0001
Service provision					
% of households with water connected to municipal network	90.0 (10.6)	91.1(9.5)	91.4 (8.0)	85.8 (14.6)	<0.0001
% of households with sewage system connected to municipal network	70.0 (25.2)	80.9 (20.7)	71.4 (23.1)	56.2 (27.3)	<0.0001
Score	−0.04 (0.4)	0.09 (0.3)	0.003 (0.3)	−0.26 (0.4)	<0.0001
**Women Empowerment measures, mean (SD)**					
Education and labor force participation					
compared to men					
Ratio female/male in education achievement for complete high school level or above ^b^	1.03 (0.1)	0.91 (0.1)	1.03 (0.1)	1.14 (0.1)	<0.0001
Ratio female/male in education achievement for complete university or above ^c^	1.07 (0.2)	0.76 (0.1)	1.08 (0.2)	1.34 (0.2)	<0.0001
Labor force participation among women ^d^	49.8 (9.7)	37.8 (4.6)	51.7 (6.9)	58.1 (5.9)	<0.0001
Ratio female/male in labor force participation^e^	0.66 (0.1)	0.51 (0.1)	0.68 (0.1)	0.75 (0.1)	<0.0001
Education achievement among women	38.4 (7.4)	32.8 (8.0)	40.3 (5.8)	40.3 (6.5)	<0.0001
% of women 25+ who completed high school or above ^f^					
% of women 25+ who completed university or above ^g^	12.7 (4.7)	9.4 (5.0)	14.3 (4.3)	12.9 (3.6)	<0.0001
Score	0.09 (0.4)	−0.24 (0.3)	0.22 (0.3)	0.15 (0.3)	<0.0001
**MMR1 coverage^h^, mean % (SD)**	91.3 (13.8)	94.3 (6.2)	90.5 (15.0)	89.9 (16.1)	0.09
**Overall Infant mortality rate^i^, mean (SD)**	11.2 (2.8)	10.9 (3.4)	11.0 (2.2)	12.0 (2.8)	0.01

a Overcrowding is defined as more than 3 people per room, excluding kitchen and bathroom in a household.

b Describes the ratio of the female to the male proportion of the population aged 25 or above who completed high school or above.

c Describes the ratio of the female to the male proportion of the population aged 25 or above who completed university or above.

d Describes % of women ≥15 years who are part of the labor force among all women ≥15 years (employed or unemployed).

e Describes the ratio between female labor force participation rate and male labor force participation rate in population older than 15 years of age.

f Describes the % female population of 25 years or above with complete high school or above among overall female population of 25 years or above.

g Describes the % female population of 25 years or above with complete university level or above among overall female population of 25 years or above.

h Describes the % of children at age 1 who received the first dose of measles-mumps-rubella vaccine among overall population at age of 1.

i Infant Mortality rate = number of infant deaths per 1000 live births.

**Table 2:** Estimated percent differences in IMRs associated with city- and country-level predictors (n = 286 cities).

	Model 1		Model2		Model 3		Model 4		Model 5	
	%	95%	%	95%	%	95%	%	95%	%	95%
	Diff	CI	Diff	CI	Diff	CI	Diff	CI	Diff	CI
Women’s labor force participation score^a^	−5.9	−20.5 11.3					−4.5	−17.5 10.4	**−6.1**	**−11.1 −0.8**
Educational attainment among women score^b^			**−9.9**	**−15.1 −4.3**			**−9.4**	**−17.5 −0.5**	−0.7	−7.0 6.2
CLE scale 2015^c^					**16.8**	**1.7 34.3**	**20.2**	**3.0 40.4**	**16.6**	**3.6 31.4**
Living conditions score^d^									**−11.9**	−18.5 −4.9
Services provision score^e^									**−10.6**	−15.8 −5.2
MMR1 coverage^f^									−0.1	−0.2 0.1
Variance	Estimate	SE	Estimate	SE	Estimate	SE	Estimate	SE	Estimate	SE
Country intercept	**0.05**	**0.02**	**0.04**	**0.02**	**0.02**	**0.01**	**0.03**	**0.01**	**0.02**	**0.004**

Null model variance, mean (SD) = 0.04 (0.02).

Note: Bold numbers are indicating statistically significant results for alpha level <0.05. Estimates corresponds to percent differences in IMRs for 1 SD higher scores of women’s labor force participation, women’s educational attainment, living conditions, and services provision; and for 1% higher MMR1 coverage and 1 point-higher CLE scale.

a Women’s labor force participation score includes the female to the male proportion of the population aged 25 or above who completed high school or above; the female to the male proportion of the population aged 25 or above who completed university degree or above; % of women ≥15 years who are part of the labor force among all women ≥15 years (employed or unemployed); ratio between female labor force participation rate and male labor force participation rate in population older than 15 years of age.

b Educational attainment among women score includes % female population with high school level or above among female population aged 25 years or above, % of female population with complete university degree or above among female population aged 25 years or above.

c Country law enforcement (CLE) scale is defined as the degree to which countries have and enforce laws supporting women’s rights, including education, family, and physical security. Scale ranges 0–4: Scale reference: (0) The laws are well enforced by the government; and is a high priority of the government; (1) laws are mostly enforced, and the government appears to be fairly proactive; (2) spotty enforcement of laws; the government may or may not signal its interest; (3) little effective enforcement; improving the situation of women appears to be a low priority for the government; (4) virtually no enforcement of laws, or such laws do not even exist.

d Living conditions Score includes % of households with piped water in the dwelling, % of households with overcrowding (3+ per room) in the house, and % of population 15-17 attending school.

e Services provision Score includes: % of households with water connected to municipal network, and % of households with sewage system connected to municipal network.

f MMR1 coverage represents the percentage of children at age 1 who received the first dose of measles-mumps-rubella vaccine among overall population at age of 1.

**Table 3:** Estimated percent differences in IMRs for city and country-level predictors by country’s GDP/capita.

	Countries below median GDP^g^			Countries above median GDP^h^			
	n = 168 cities			N = 118 cities			Test for interaction*
	% difference	95% CI		% difference	95% CI		p-value
Women’s labor force participation score^a^	**−5.8**	**−10.0**	**−1.4**	**−14.6**	**−17.6**	**−11.5**	0.26
Educational attainment among women score^b^	**−3.6**	**−6.3**	**−0.9**	7.8	−3.1	20.0	**0.02**
CLE score 2015^c^	34.0	−13.9	108.5	13.7	−29.0	82.0	0.32
Living conditions score^d^	**−15.1**	**−22.0**	**−7.4**	**−12.9**	**−16.7**	** −9.0**	**0.03**
Services provision score^e^	**−12.6**	**−13.5**	**−11.7**	4.9	−5.1	16.0	**<0.0001**
MMR1 coverage^f^	−0.1	−0.1	0.01	−0.2	−0.9	0.6	0.91

Note:Bold numbers are indicating statistically significant results for alpha level <0.05. Estimates corresponds to percent differences in IMRs for 1 SD higher scores of women’s labor force participation, women’s educational attainment, living conditions, and services provision; and for 1% higher MMR1 coverage and 1 point-higher CLE scale.

* Each interaction was tested separately along with the full model.

a Women’s labor force participation score includes the female to the male proportion of the population aged 25 or above who completed high school or above; the female to the male proportion of the population aged 25 or above who completed university degree or above; % of women ≥15 years who are part of the labor force among all women ≥15 years (employed or unemployed); ratio between female labor force participation rate and male labor force participation rate in population older than 15 years of age.

b Educational attainment among women score includes % female population with high school level or above among female population aged 25 years or above, % of female population with complete university degree or above among female population aged 25 years or above.

c Country law enforcement (CLE) scale is defined as the degree to which countries have and enforce laws supporting women’s rights, including education, family, and physical security. Scale ranges 0–4: Scale reference: (0) The laws are well enforced by the government; and is a high priority of the government; (1) laws are mostly enforced, and the government appears to be fairly proactive; (2) spotty enforcement of laws; the government may or may not signal its interest; (3) little effective enforcement; improving the situation of women appears to be a low priority for the government; (4) virtually no enforcement of laws, or such laws do not even exist.

d Living conditions Score includes % of households with piped water in the dwelling, % of households with overcrowding (3+ per room) in the house, and % of population 15–17 attending school.

e Services provision Score includes: % of households with water connected to municipal network, and % of households with sewage system connected to municipal network.

f MMR1 coverage represents the percentage of children at age 1 who received the first dose of measles-mumps-rubella vaccine among overall population at age of 1.

g Countries with GDP/capita below the median (15,530.7 US$) are Brazil, Colombia, Costa Rica, and Peru.

h Countries with GDP/capita above the median (15,530.7 US$) are Argentina, Chile, Mexico, and Panama.
