# Supplementary material for: Women’s empowerment and infant mortality in Latin America: evidence from 286 cities
Source: Cities Health. 2021 Apr 22;7(1):93–101. doi: 10.1080/23748834.2021.1908794 (PMC7614198; doi:10.1080/23748834.2021.1908794)
Supplement: Resumen Espanol & Resumo Portugues 1908794 [file RCAH_A_1908794_SM0597.pdf]

Ana Ortigoza, Ariela Braverman, Philipp Hessel, Vanessa Di Cecco, Amélia Augusta Friche, Waleska Teixeira Caiaffa & Ana V. Diez Roux (2021) Women’s empowerment and infant mortality in Latin America: evidence from 286 cities, Cities & Health, DOI: 10.1080/23748834.2021.1908794

RESUMEN

Los niveles de empoderamiento de las mujeres (EM) pueden contribuir a las diferencias en las tasas de mortalidad infantil (TMI) entre ciudades. Utilizamos un estudio de corte-transversal multinivel para examinar las asociaciones de EM y TMI en 286 ciudades de siete países en América Latina. Estimamos las TMI para el período 2014-2016 y combinamos indicadores socioeconómicos de las ciudades para crear factores que reflejan las condiciones de vida y la prestación de servicios. El EM se caracterizó : (1) a nivel de ciudades, mediante el uso de puntajes que reflejan la participación de las mujeres en la fuerza laboral (PMFL) y el logro educativo entre las mujeres, derivados de indicadores de educación y empleo desagregados por sexo; (2) a nivel de los países, través del uso de una escala que mide el nivel de implementación de leyes relacionadas con los derechos de las mujeres. Estimamos las diferencias porcentuales ajustadas en las TMI asociadas que se asocian a niveles de EM más altos, en el total de las ciudades y estratificadas por el PIB del país. Encontramos una heterogeneidad sustancial en las TMI y los niveles de EM a nivel de las ciudades. Un nivel de PMFL más alto se asoció con TMI más bajas. Niveles más altos en el logro educativo de las mujeres se asoció con TMI más bajas únicamente en ciudades de países con un PIB más bajo. La implementación nacional más deficiente de leyes que protegen los derechos de las mujeres se asoció con TMI más altas en todos los países. El empoderamiento de las mujeres podría tener implicaciones positivas para la salud de la población. Fomentar el desarrollo socioeconómico de las mujeres y la educación de las niñas debe ser parte de las estrategias para reducir las TMI en las ciudades del Sur Global.

RESUMO

Os níveis de empoderamento das mulheres (EM) podem contribuir para as diferenças nas taxas de mortalidade infantil (TMI) entre as cidades. Usamos um estudo transversal multinível para examinar as associações de EM com TMI em 286 cidades em sete países da América Latina. Estimamos as TMI para o período de 2014–2016 e combinamos os indicadores socioeconômicos da cidade em fatores que refletem as condições de vida e a prestação de serviços. O EM foi operacionalizado: (1) nas cidades, usando pontuações para participação das mulheres na força de trabalho (PMFT) e escolaridade entre mulheres, por meio de indicadores de educação e emprego desagregados por sexo; (2) nos países, utilizando uma escala que mensura o nível de implementação de leis relacionadas aos direitos das mulheres. Estimamos diferenças percentuais ajustadas das TMI associadas a pontuações de EM mais altas em todas as cidades e estratificadas pelo PIB do país. Encontramos heterogeneidade substancial das TMI e EM nas cidades. O PMFT (WLFP) mais alto foi associado a TMI mais baixas. O nível educacional mais alto das mulheres foi associado a TMI mais baixas apenas em cidades de países com PIB mais baixo. A implementação nacional mais ineficiente das leis que protegem os direitos das mulheres foi associada a TMI mais altas em todos os países. O empoderamento das mulheres pode ter implicações positivas para a saúde da população. A promoção do desenvolvimento socioeconômico das mulheres e a educação das meninas devem fazer parte das estratégias para reduzir as TMI nas cidades do Sul Global.

PALABRAS CLAVE: empoderamiento de la mujer; mortalidad infantil; America latina

PALAVRAS-CHAVE: empoderamento das mulheres; mortalidade infantil; América latina

Information Classification: General
