# Supplementary material for: Women’s empowerment and infant mortality in Latin America: evidence from 286 cities
Source: Cities Health. 2021 Apr 22;7(1):93–101. doi: 10.1080/23748834.2021.1908794 (PMC7614198; doi:10.1080/23748834.2021.1908794)
Supplement: Supplemental Material [file RCAH_A_1908794_SM5495.pdf]

Supplementary material

Table 1. Socio economic characteristics of cities included and excluded in the analysis

	Total n (% of sample) or mean (SD)	Included in the sample	Excluded from the sample	p-value
**Overall number of cities n (%)**	360	286 (79·4%)	74 (20·5%)	
**Socio-economic predictors by domain mean, (SD)**				
Living conditions				
% of households with piped water in the dwelling	87·1 (13·3)	89·7 (11·5)	77·0 (15·2)	<0·0001
% of households with overcrowding in the house [a]	5·8 (4·6)	4·9 (3·9)	9·2 (5·1)	<0·0001
% of population 15-17 attending school	79·1 (7·9)	80·4 (7·5)	74·6 (7·7)	<0·0001
Score	0·002(0·7)	0·14 (0·6)	-0·51(0·6)	<0·0001
Services provision				
% of households with water connected to municipal network	88·8 (12·0)	90·0 (10·5)	84·4 (15·7)	<0·0001
% of households with sewage system connected to municipal network	70·2 (24·4)	69·9 (25·2)	70·6 (21·5)	0·84
Score	0·002 (0·4)	0·03(0·4)	-0·08(0·4)	0·05
**Women's Empowerment measures, mean (SD)**				
Education and labor force participation compared to men				
Ratio female/ male in education achievement for complete high school level or above [b]	1·01 (0·1)	0·95(0·1)	1·02 (0·1)	<0·0001
Ratio female/ male in education achievement for complete university or above [c]	1·03 (0·2)	0·91 (0·2)	1·07(0·2)	<0·0001
Labor force participation among women [d]	48·1 (10·0)	41·0 (7·6)	49·8 (9·7)	<0·0001
Ratio female/male in labor force participation [e]	0·64 (0·1)	0·56 (0·1)	0·66 (0·1)	<0·0001
Score	0·15 (0·8)	-0·38 (0·7)	0·29 (0·8)	<0·0001

	Total n (% of sample) or mean (SD)	Included in the sample	Excluded from the sample	p-value
Educational attainment among women				
% of women 25+ who completed high school or above [f]	37·3 (8·0)	33·1 (9·0)	38·4 (7·3)	<0·0001
% of women 25+ who completed university or above [g]	12·8 (5·0)	13·1 (6·1)	12·7 (4·8)	0·56
Score	0·15 (0·4)	0·04 (0·4)	0·17 (0·3)	0·01
**MMR1 coverage [h], mean % (SD)**	11.3 (3·1)	11·2 (2·7)	11·7 (4·3)	0·32
**Overall Infant mortality rate [i], mean (SD)**	90·9 (14·2)	91·2 (13·7)	89·5 (15·8)	0·28

(a) Overcrowding is defined as more than 3 people per room, excluding kitchen and bathroom in a household
(b)Describes the ratio of the female to the male proportion of the population aged 25 or above who completed high school or above
(c) Describes the ratio of the female to the male proportion of the population aged 25 or above who completed university or above
(d) Describes % of women ≥ 15 years who are part of the labor force among all women ≥ 15 years (employed or unemployed)
(e) Describes the ratio between female labor force participation rate and male labor force participation rate in population older than 15 years of age
(f) Describes the % female population of 25 years or above with complete high school or above among overall female population of 25 years or above
(g) Describes the % female population of 25 years or above with complete university level or above among overall female population of 25 years or above
(h) Describes the % of children at age 1 who received the first dose of measles-mumps-rubella vaccine among overall population at age of 1.
(i) Infant Mortality rate = number of infant deaths per 1,000 live births
